# Carbonaceous Aerosols in Fine Particulate Matter of Santiago Metropolitan Area, Chile

**DOI:** 10.1155/2014/794590

**Published:** 2014-01-22

**Authors:** Richard Toro Araya, Robert Flocchini, Rául G. E. Morales Segura, Manuel A. Leiva Guzmán

**Affiliations:** ^1^Centro de Ciencias Ambientales et Departamento de Química, Facultad de Ciencias, Universidad de Chile, Las Palmeras 3425, Ñuñoa, 7800003 Santiago, Chile; ^2^Department of Land, Air and Water Resources (LAWR), University of California, One Shields Avenue, Davis, CA 95616, USA

## Abstract

Measurements of carbonaceous aerosols in South American cities are limited, and most existing data are of short term and limited to only a few locations. For 6 years (2002–2007), concentrations of fine particulate matter and organic and elemental carbon were measured continuously in the capital of Chile. The contribution of carbonaceous aerosols to the primary and secondary fractions was estimated at three different sampling sites and in the warm and cool seasons. The results demonstrate that there are significant differences in the levels in both the cold (March to August) and warm (September to February) seasons at all sites studied. The percent contribution of total carbonaceous aerosol fine particulate matter was greater in the cool season (53 ± 41%) than in the warm season (44 ± 18%). On average, the secondary organic carbon in the city corresponded to 29% of the total organic carbon. In cold periods, this proportion may reach an average of 38%. A comparison of the results with the air quality standards for fine particulate matter indicates that the total carbonaceous fraction alone exceeds the World Health Organization standard (10 *µ*g/m^3^) and the United States Environmental Protection Agency standard (15 *µ*g/m^3^) for fine particulate matter.

## 1. Introduction

Carbonaceous aerosols (CAs) are an important constituent of breathable atmospheric aerosol particulate matter mass (PM). CA particles have aerodynamic diameters less than 2.5 microns (PM_2.5_) and account for as much as 40% of the PM_2.5_ mass in cities worldwide [1, 2]. These particulates are associated with serious human health problems, including respiratory, cardiovascular, and cerebrovascular diseases, affecting morbidity and mortality levels [3]. CAs also have an impact on ecosystems [4]. For these reasons, CAs have become an important and active research topic in the last decade [5].

CA can be characterized by its organic carbon (OC) and elemental carbon (EC) content [6]. OC can be directly emitted to the atmosphere in particulate form (primary organic aerosols, POCs) [7], or it can have a secondary origin from gas-to-particle conversion of volatile organic compounds in the atmosphere [8]. In general, OC is a mixture of hydrocarbons and oxygenated compounds, including polycyclic aromatic hydrocarbon and other organic compounds with possible mutagenic and carcinogenic effects [3, 4]. On the other hand, the EC fraction is essentially a primary pollutant, emitted directly during the incomplete combustion of fossil and biomass carbonaceous fuels [7].

**Figure 1:**
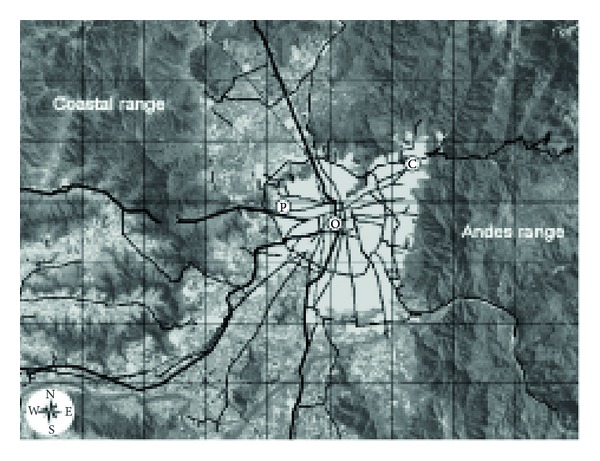
Regional topography of Santiago Metropolitan Area (SMA), Chile (with 10 km grid lines). The gray area is the urban region, and the black lines represent the main routes and streets. White dots designate the three air quality monitoring stations of the MACAM-2 network that were used in this study (C: Las Condes, O: Parque O'Higgins, and P: Pudahuel).

The number, size, and geographical distribution of large urban centers have increased dramatically during the second half of the 20th century [9]. These concentrations of people and activity are exerting increasing stress on the natural environment, with impacts at local, regional, continental, and global scales. In this sense, the population in the Santiago Metropolitan Area (SMA) of Chile (33.5° S, 70.6° W; see Figure 1) has grown from about four hundred thousands in the 1940s to six million and half in 2010 [10], soon to become in a megacity, and air pollution has been and is one of the most important environmental problems in the city and in general in cities at global level.

Today, Santiago, the capital of Chile, is the most important urban center in the country and the fifth largest city in South America [9]. The population of Santiago represents 40% of the country's population [11]. SMA is the main financial, cultural, commercial, industrial, and political center of Chile and has one of the highest annual mean PM_2.5_ concentrations when compared with other cities throughout the world [12].

Seguel et al. [13] used an EC tracer technique to investigate the daytime secondary organic aerosols (SOA) variation from 10:00 to 18:00 local time in Santiago over one month by conducting real-time measurements of organic and elemental carbon. They estimated that the contribution of SOA to OC could reach 20% of total organic aerosol matter in the summer (February 2004) in Santiago. In a recent study, organic aerosols were analyzed by applying positive matrix factorization (PMF) for organic mass spectra [14]. The results demonstrated that the aerosol particles were composed primarily of organics (59%) and that the sources were related to fresh automobile exhaust and biomass burning. In general, these studies were limited to short-term measurements. Several other studies of the chemical composition of PM_2.5_ have been performed in Santiago, but the relationship between the formation of SOA and the sources of OC and EC has not been thoroughly investigated.

In the present work, to better understand SOA formation and the sources of OC and EC, we examined the OC and EC content in PM_2.5_ data collected from the Air Quality Monitoring Network of the Metropolitan Area of Santiago (MACAM, Spanish acronym) over a period of 6 years (2002–2007). The aims of the study were to (i) characterize the OC and EC concentrations in an urban atmosphere, (ii) augment the knowledge of spatial and seasonal variations of PM_2.5_ associated with EC and OC concentrations, and (iii) estimate the seasonal and spatial trends of secondary OC (SOA) contributions to total carbonaceous loading.

## 2. Materials and Methods

### 2.1. Sampling Sites and Descriptions

The city of Santiago (33.5 S, 70.6 W) is located in a valley in the central zone of Chile between the Maipo and Mapocho rivers, with a geographic area of approximately 1400 km^2^. It is 500 m above sea level and is surrounded by the Andes and Coastal mountains (Figure 1). The climate in SMA is classified as Mediterranean; although its wind patterns are complex because of the topography and urban surface roughness, SMA is characterized by a very persistent valley-mountain breeze system with a predominant low-speed (frequently lower than 2.0 ms^−1^) wind from the southwest in autumn and winter (Morales, 2006). In addition, the prevailing anticyclonic meteorological conditions throughout the year lead to a permanent subsidence and thermal inversion layer between 400 and 1000 m above the city, thus providing a very stable atmospheric gradient that reduces the dispersion of air pollutants [15].

Eight monitoring stations distributed around the city measure concentrations of criteria atmospheric pollutants and collect meteorological data (MACAM-2 network) [16]. Of the stations, only three record OC and EC: Las Condes (C label), Parque O'Higgins (O label), and Pudahuel (P label) (see Figure 1 and Table 1). The data from those stations were used in this study.

Parque O'Higgins station (O) measures the air quality in the downtown area of the city. The station is located in a large park approximately 2 km south of the city center and 1 km west of a major highway, which has traffic of approximately 60,000 vehicles per day. The area features a mixture of houses, retail, and light industry. The Pudahuel (P) sampling site is located in a small park near a medical clinic in the western part of SMA. This area has two major roads that support the commercial activity: one road runs toward the south and has traffic of approximately 20,000 vehicles per day, and the other is in the west and has traffic of approximately 15,000 vehicles per day. Most of this area is residential, but Pudahuel also features agricultural areas, an international airport, wetlands, and the largest sewage-treatment plants in SMA. The Las Condes (C) station is located in the eastern part of SMA. It is in a small park south of a street with traffic of approximately 15,000 vehicles per day. The area is primarily residential.

### 2.2. Pollutant Concentration Measurement

The PM_2.5_ ambient concentration was measured with tapered element oscillating microbalance (TEOM) monitors from Rupprecht & Patashnick Co., Inc. (Albany, New York). The instrument uses an oscillating hollow tube whose free end is attached to a filter element. As particles accumulate, the filters mass changes and the oscillating frequency changes, thereby providing a measurement of the mass. The tapered tube, filter, and sampled air were kept at 50°C. The sampling interval was set to 15 min.

The OC and EC ambient concentrations in PM_2.5_ were continuously determined by a thermal analysis method using Model 5400 ambient carbon particulate monitors manufactured by Rupprecht & Patashnick Co., Inc. (RPM5400). This instrument collects airborne particulate matter (PM_2.5_) at a constant temperature of 50°C and constant flow (1 m^3^ h^−1^) on an impactor plate with a 50% cut-off diameter of 0.14 *μ*m. Next, a sequential oxidation is produced in particle-free ambient air at different combinations of temperature to pyrolyze and combust the carbon-containing compounds, which are then measured with a CO_2_ nondispersive infrared sensor. The amount of carbonaceous substances evolved at 340°C was defined as OC and the corresponding amount at 750°C as total carbon (TC).

The quality assurance and quality control program for this type of measurements consist in a check of the flow rate at the inlet with an audit flow meter and automatic leak check and CO_2_ audit (zero and span) when bottled N_2_ and CO_2_ span gas are connected to the monitor. This performance is operated on a weekly basis. The data obtained from the MACAM network were validated for fix vacancies, duplicated entries, and gaps.

### 2.3. Statistical Data Analysis

Statistical data analysis was carried out in the open source statistical software R programming language (R Development Core Team, 2012) and its packages OpenAir under RStudio: integrated development environment (Version 0.97.551) (Computer software), Boston, MA. RStudio is available from http://www.rstudio.org/. The OpenAir website at http://www.openair-project.org/ provides more information concerning the project and a comprehensive manual that supports the package and references.

### 2.4. EC Tracer Method

The direct separation and quantification of POC and SOC is difficult, primarily because of the complexity of the OC reaction pathways, the vast number of products formed by photochemical and thermal oxidation reactions, and the cost involved in the analytical methods required for speciation. However, the EC tracer method is a semiempirical method for quantitative assessment of SOC that has become very useful [13, 17–20]. The EC tracer approach suggests that samples with the lowest OC/EC ratio are almost exclusively POC. Thus, the SOC fraction is estimated by using EC as a tracer and assuming that POC can be obtained from

(1) $$\begin{array}{c} \text{POC}=\text{EC}\times {\left(\frac{\text{OC}}{\text{EC}}\right)}_{\text{pri}}+\text{O}{\text{C}}_{\text{noncomb}}. \end{array}$$

Then, the SOC contribution to the total OC can be estimated as the difference between the total OC and POC concentrations:

(2) $$\begin{array}{c} \text{SOC}=\text{O}{\text{C}}_{\text{total}}-\text{POC}, \end{array}$$

where (OC/EC)_pri_ is the estimated primary carbon ratio and OC_noncomb_ is the noncombustion OC from biogenic POC and other sources (i.e., meat cooking) [13, 17, 20]. This OC_noncomb_ fraction could also include contributions from sampling artifacts or the regional OC background. Several methods to determine (OC/EC)_pri_ have been proposed in the literature. The objective of each of the methods is to determine the minimum OC/EC ratio, that is, (OC/EC)_min⁡_, of a given period, which is determined according to the temporal resolution of the available carbon concentrations. Some authors have proposed that [21], when available, daily concentrations yield the lowest 2.5% of measured OC/EC values in a given month and/or season [21]. Moreover, when hourly concentrations are available, (OC/EC)_min⁡_⁡ is obtained from a linear relationship between the OC and EC of a given period, thereby distinguishing emissions scenarios and/or environmental conditions that may occur during the measurements. In this work, we used a linear relationship between the hourly values of OC and EC and included only the values of the daily minimum OC/EC ratio. This dataset yielded a linear relationship, whose regression was used to determine SOC and POC according to (1) and (2), respectively.

## 3. Results and Discussion

### 3.1. Concentrations of PM_2.5_, OC, and EC

**Figure 2:**
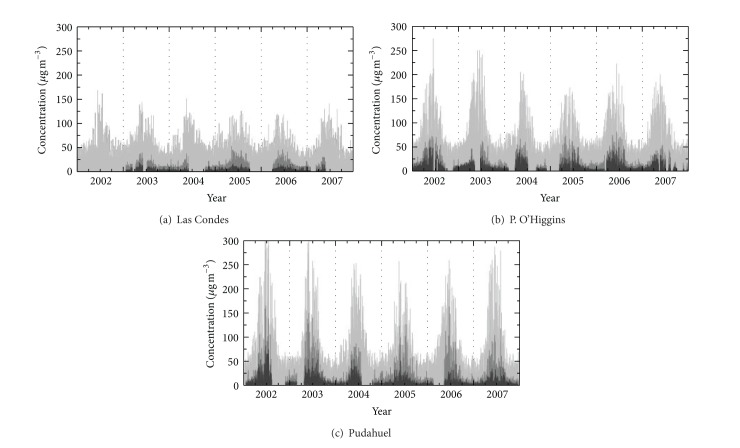
Hourly mass concentration (in *μ*g/m^3^) time series of PM_2.5_ (light gray), organic carbon (OC, gray), and elemental carbon (EC, dark gray) in PM_2.5_ at the (a) Las Condes (labeled C), (b) Parque O'Higgins (labeled O), and (c) Pudahuel (labeled P) stations. The station labels are the same as in Figure 1. Data source: National Information System of Air Quality, Chilean Ministry of the Environment, Chile.

The average hourly concentrations of PM_2.5_, OC, and EC recorded at the three sampling sites studied (C, O, and P; see Figure 1) from 2002 to 2007 are plotted in Figure 2. Statistical data regarding the mass concentration in *μ*g/m^3^ of PM_2.5_, OC, EC, and total carbonaceous aerosols (TCA) at the three sampling sites are presented in Table 2. The hourly PM_2.5_ mass concentrations ranged from 0.1 to 425 *μ*g/m^3^, with 1% and 99% percentiles of 3.3 *μ*g/m^3^ and 136 *μ*g/m^3^, respectively. The hourly OC and EC mass concentration averages for the three monitoring stations during the sampling period were 10.5 ± 10.4 *μ*g/m^3^ and 3.39 ± 4.96 *μ*g/m^3^, respectively. The OC levels were always higher than EC at all sampling sites (Figure 2). The hourly mass concentrations ranged from 0.01 to 164 *μ*g/m^3^ for OC and 0.04 to 80.7 *μ*g/m^3^ for EC. The 1% and 99% percentiles for OC and EC were 1.60 *μ*g/m^3^–52.4 *μ*g/m^3^ and 0.10 *μ*g/m^3^–24.7 *μ*g/m^3^, respectively. TCA values were calculated as the sum of EC and organic matter (OM), which was estimated to be 1.6 times OC [20]; see Table 2. The hourly TCA concentrations ranged from 0.14 to 177.5 *μ*g/m^3^, and the mass concentration average was 15.5 ± 14.7 *μ*g/m^3^. The TCA/PM_2.5_ ratios could reflect the source apportionment of carbonaceous fractions in terms of PM mass. TCA represented an annual average of almost 48 ± 41% of PM_2.5_.

The concentrations of OC, EC, and TCA exhibited seasonal trends similar to that of PM_2.5_. In general, the concentrations of OC, EC, and PM_2.5_ exhibited minima in the spring-summer (September to February), that is, warm seasons, and maxima in winter-autumn (March to August), that is, cool seasons, at all the monitoring sites studied (see Table 2). The wintertime OC and EC concentrations were approximately 2 to 5 times the corresponding concentrations during the summer. High PM_2.5_, OC, and EC concentrations in the cool seasons are the result of increased emissions from heating sources and the prevalence of Pacific anticyclonic meteorological conditions, which feature a permanent subsidence and thermal inversion layer with a mixing height of approximately 400 m [15]. In the warm seasons, better dispersion conditions provided by an increased boundary layer height (1000 m) and a diurnal mountain-valley breeze contribute to the dispersal of primary emissions of gases and PM_2.5_ [15]. The percent contribution of TCA to PM_2.5_ was greater for the cool seasons (53 ± 41%) than for the warm seasons (44 ± 18%). The greater contribution of TCA in the cool seasons may be attributed to more significant emission sources of carbonaceous aerosols and meteorological factors (low temperature and low wind speed). The average fraction of TCA in PM_2.5_ was similar to that in cities worldwide (e.g., 45.6% in Xiamen, China [22]; 35.1% in Tianjin, China [23]; and 46.5% in Madrid, Spain [24]).

Seasonal and site differences in PM_2.5_, OC, and EC concentrations were tested by one-way analysis of variance (ANOVA) implemented in the statistical software package MS Excel ©. The results of the analysis are shown in Table 3. No significant (*P* > 0.05) yearly variability in PM_2.5_, OC, and EC at the three stations studied was found by *F*-test (which might indicate that the OC and EC source emission rates, such as vehicle exhaust, were consistent at these sites during the years studied). Significant (*P* < 0.05) seasonal variability in PM_2.5_, OC, and EC was found for the three sites studied. The obvious seasonal variations in PM_2.5_, OC, and EC found at these sites are produced mainly as a result of the accumulation of emissions because of the low mixing height during the winter season and the influence of increased local sources from heating (Morales, 2006).

PM_2.5_, OC, and EC concentrations can vary from site to site. Our results demonstrate that the mean OC and EC concentrations in PM_2.5_ were highest in urban-industrial areas and lowest in urban-suburban areas. The Pudahuel station (Figure 2(c)) is located at an urban site that is influenced by local primary emissions from nearby industrial, international airport, and residential areas in the western part of the city. The lowest mean OC and EC concentrations occurred at the Las Condes station (Figure 2(a)), which is located in an exclusively residential area in the eastern part of the city. Consequently, significant differences (*P* < 0.05) in the PM_2.5_, OC, and EC concentrations were observed among the sites studied.

**Figure 3:**
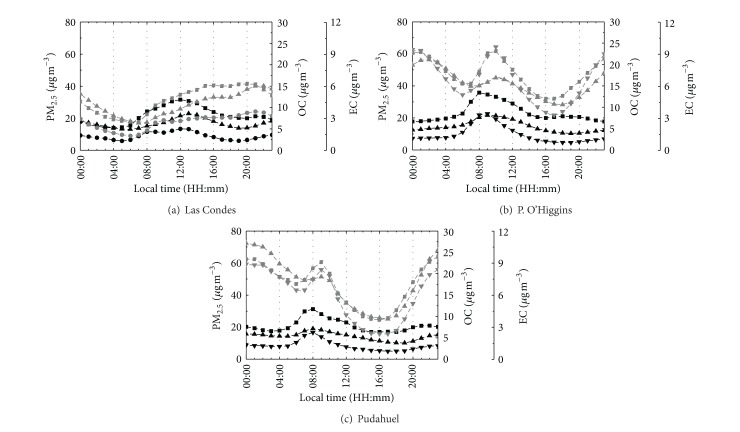
Daily mass concentration (in *μ*g/m^3^) in the cool (gray) and warm seasons (black) of PM_2.5_(black square, grey square), organic carbon (OC; grey triangle), and elemental carbon (EC; inverted triangle) in PM_2.5_ at the (a) Las Condes (labeled C), (b) Parque O'Higgins (labeled O), and (c) Pudahuel (labeled P) stations in the cool (gray line) and warm (black line,) seasons. Label of the stations in Figure 1: Las Condes: C Label, P. O'Higgins: O Label, and Pudahuel: P Label. Data source: Chilean Ministry of Health, Metropolitan area (SEREMI-RM).

Figure 3 presents the diurnal variations in OC, EC, and PM_2.5_ in SMA in the cool and warm seasons. In the cool seasons, the PM_2.5_, OC, and EC concentrations were greater at night than during the day. In general, PM_2.5_, EC, and OC exhibited two peaks during the study: one in the morning at 8:00–10:00 and one in the late evening at 20:00–23:00. However, at Las Condes station, a maximum occurred in the late evening that is associated with airborne material transported by the wind that follows the typical diurnal southwest predominant wind direction observed in the city. This peak can also be attributed to local mobile sources because it is consistent with rush-hour traffic in afternoon and can be attributed to increased wood burning at night. In addition, the daily variations in the concentrations of PM_2.5_, OC, and EC were strongly affected by diurnal variations in the mixing height. PM_2.5_, OC, and EC concentrations were low during the day when the mixing height was higher (400 m in the cool season) (Morales, 2006); this effect dilutes the particle pollution released at the surface and results in a lower ambient concentration. However, the concentrations were high during the night when the mixing height was less than 100 m (Morales, 2006) in the cool season, when poor dispersion conditions prevailed. Otherwise, in warm seasons, PM_2.5_, OC, and EC exhibited a clear early-morning peak (6:00–10:00), which is consistent with rush-hour traffic in the morning. In the warm seasons, the mixing height is higher than in the cold seasons, and wood burning for heating is reduced; therefore, a maximum concentration at night was not observed.

**Figure 4:**
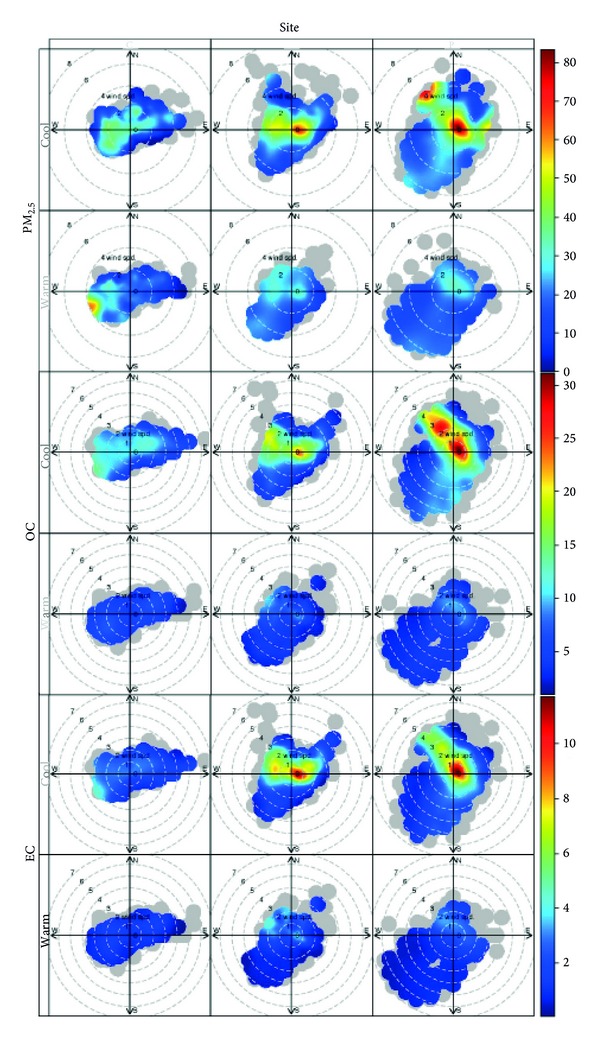
Bivariate polar plot for the mean concentration of PM_2.5_, OC, and EC for the sites under study in cold (winter and fall seasons) and warm periods (summer and spring seasons). Label of the stations in Figure 1. Las Condes: C Label, P. O'Higgins: O Label, and Pudahuel: P Label.

Figure 4 shows the bivariate polar plot for the mean concentration of PM_2.5_, OC, and EC for the sites under study, in warm and cold periods. Winds from the east-west are observed in the cold period in the C site. These winds correspond to the valley-mountain breeze pattern. Higher levels of concentration of PM_2.5_, OC, and EC are observed in calm winds conditions and when the winds blow from the downtown. These high levels can be explained by local emission and transport from the city downtown. At the site O, concentrations of PM_2.5_, OC, and EC are notoriously higher in cold periods and occur preferentially when the wind speed is less than 2 m/s. The O site is located approximately in the center of the valley of SMA and also shows the mountain-valley breeze although weaker than site C. On this site the highest concentrations of OC and EC are produced when the wind speed is low (as well as PM_2.5_). However, in cold periods maximum concentrations also occur with winds >3 m/s indicating that at site O there is also a contribution due to transport of carbon particles from the east. Site P produced the highest concentrations of PM_2.5_, OC, and EC in cold periods when the wind speed is less than 1 m/s. This indicates that the contribution of local sources could be more significant in this site. During the cold periods, a similar behavior for PM_2.5_, OC, and EC was found (winds < 2 m/s). In addition, peak concentrations for PM_2.5_ and OC were observed when the wind exceeds 2 m/s from the northwest. This shows a particular behavior for the P site, where high concentration events of PM_2.5_ can occur in two scenarios, one dominated by local sources and another produced by the transport of polluted air masses from the northwest in which the organic fraction can be significant.

### 3.2. Correlation between OC and EC and the OC/EC Ratio

**Figure 5:**
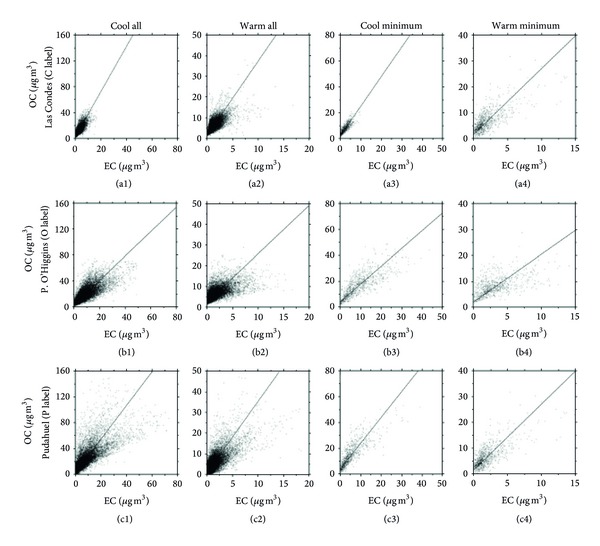
Scatter plot of OC and EC in PM_2.5_, corresponding to the hourly cool and warm season datapairs, for the period from 2002 to 2007 at the three stations studied. Figures i1 and i2 (i = a, b and c) show scatter plots of all data available for the years of the study in a cool (1) and warm (2) seasons of all years. Figures i3 and i4 (i = a, b and c) show scatter plots of the minimum OC/EC ratio in the cool (3) and warm (4) seasons during the years of the study. Label of the stations in Figure 1. Las Condes: C Label, P. O'Higgins: O Label, and Pudahuel: P Label.

The OC versus EC correlation during the cool and warm seasons studied is shown in Figure 5. The data were modeled by linear regression; the results are given in Tables 4 and 5. The intercept (a) is interpreted as the OC background concentration that originates from noncombustion sources, although it may be biased by uncertainty in carbon measurements and by the relatively large slope. The OC/EC slope, or b value, for all data available in the cool and warm season at each station (Figures 5(a1), 5(a2), 5(b1), 5(b2), 5(c1), and 5(c2)) in Table 4 varies from 1.30 to 2.48, with an average of 1.79 ± 0.43. Similar values were reported for Hong Kong (1.9) [25], Los Angeles (2.0) [17], Helsinki (2.5) [26], Guangzhou (2.5) [27], and Beijing (2.9) [28].

Several investigators have used the OC/EC ratio to obtain some indication of the origins of carbonaceous PM_2.5_ [29]; if the major fractions of OC and EC are emitted by a dominant primary source (e.g., residential and commercial coal combustion or motor vehicle exhaust), the correlation between the OC and EC concentrations should be high because the relative rates of EC and OC emissions would be proportional to each other. A strong OC/EC correlation (*R* ^2^: 0.62–0.68) was observed for the cool seasons, which suggests impacts from a combination of common sources (Table 4). By contrast, in the warm seasons, the OC/EC correlations were lower than those in the cool seasons (*R* ^2^: 0.44–0.49) and were scattered. This result suggests that the OC and EC emissions in warm seasons may not be released from a single dominant primary source, possibly due to the impact of sources that are unrelated to local vehicular emissions (i.e., SOC).

As indicated above, the POC content (OC/EC)_prim_ can be obtained from the minimum OC/EC ratio observed in atmospheric samples that contain exclusively primary carbonaceous compounds; see Figures 5(a3), 5(a4), 5(b3), 5(b4), 5(c3), and 5(c4). The minimum OC/EC ratios at the 3 sampling sites in the cool and warm seasons are presented in Table 5. The observed minimum value of the OC/EC ratio in this study ranged from 1.20 ± 0.02 to 1.92 ± 0.05 in the cool season and from 1.15 ± 0.03 to 1.43 ± 0.04 in the warm season. These values were within the range of 0.9–2.0 times the reported values for primary aerosols but equal to the minimum OC/EC ratios of, for example, 1.7 in Beijing [2], 1.5 in Kaohsiung City [30], 1.1 in Birmingham [31], and 0.8–1.0 in Saporo and Uji [27].

### 3.3. Primary and Secondary Source Contributions

From (1) and (2) and the linear regression constants presented in Table 5, it is possible to estimate the POC and SOC contributions to the organic fraction. This estimation was performed for the warm and cool seasons during the period from 2002 to 2007 and the results are presented in Table 6.

The annual average concentrations of SOC and its contribution to ambient OC were estimated to be 1.6 *μ*g/m^3^ (25.2%), 1.3 *μ*g/m^3^ (23.7%), and 1.6 *μ*g/m^3^ (28.8%) in warm seasons and 3.2 *μ*g/m^3^ (29.6%), 4.2 *μ*g/m^3^ (26.8%), and 6.8 *μ*g/m^3^ (37.6%) in cool seasons at C, O, and P sites, respectively, during the period from 2002 to 2007. The seasonally averaged values demonstrated that the SOC proportion of the OC was slightly greater during cool seasons than warm seasons at all stations studied. These results suggest that SOCs may be a significant contributor to fine organic particles such as OC throughout the year. In warm seasons, SOCs may contribute to OC under meteorological conditions favorable to the occurrence of photochemical activity, and in cool seasons, they may contribute to OC under high pollution conditions and sometimes accompanied by cloudless days with high photochemical activity.

SOA is an important determinant of the physical and chemical properties of the atmosphere related to haze, visibility, climate, and health. The annual average concentrations of SOA estimated at the 3 sampling sites in SMA ranged from 2.1 to 2.5 and from 5.1 to 10.9 *μ*g/m^3^ for the warm and cool seasons, respectively, thereby accounting for 9.0–23.4% of PM_2.5_ mass, which indicates that SOA also contributes a major fraction of the PM_2.5_ mass in SMA.

The estimations of SOC in this research are in agreement with those in urban areas. For example, in Santiago, in our previous work [13], we observed a SOC value as great as 20% of the total organic aerosol matter in February 2004. In some areas of Madrid, Spain [24], and Milan, Italy [32], SOC represents between 35% and 24% of total organic aerosol matter. In the Pearl River Delta Region, China, SOC accounts for 43% of organic carbon concentrations in winter in PM_2.5_ [33].

### 3.4. PM_2.5_, Carbonaceous Fraction, and Human Health Impact

Many epidemiological studies have demonstrated a statistical association between airborne aerosols and premature death and adverse cardiovascular effects, including increased hospitalizations and emergency department visits for heart attacks and strokes [3, 4]. The mechanism by which fine particles cause disease and death is unknown. It has been suggested that particles retained deep in the lungs cause inflammation, which, in turn, releases bioactive substances into the bloodstream, thereby causing coagulation. The release of bioactive substances is also caused by organic compounds.

To protect human health from the known effects of PM, organizations such as the World Health Organization (WHO), the United States Environmental Protection Agency (EPA), and the Environmental Ministry of Chile (MMA) have established safety thresholds for environmental concentrations of PM_10_ and PM_2.5_. The annual average PM_2.5_ mass concentration was 32 *μ*g/m^3^ during the sampling period at the 3 monitoring stations; this value is almost 1.6, 2.2, and 3.2 times higher than the Chilean standard (20 *μ*g/m^3^) [34], the EPA standard (15 *μ*g/m^3^) [35], and the WHO guideline (10 *μ*g/m^3^) [36], respectively. Even more, the average total carbonaceous aerosol represents for the whole study period at the three sites 48% of PM_2.5_ (43% in warm periods and 52% in cold periods). Therefore, in the city of Santiago this fraction by itself exceeds thresholds for fine particles of the WHO and EPA.

**Figure 6:**
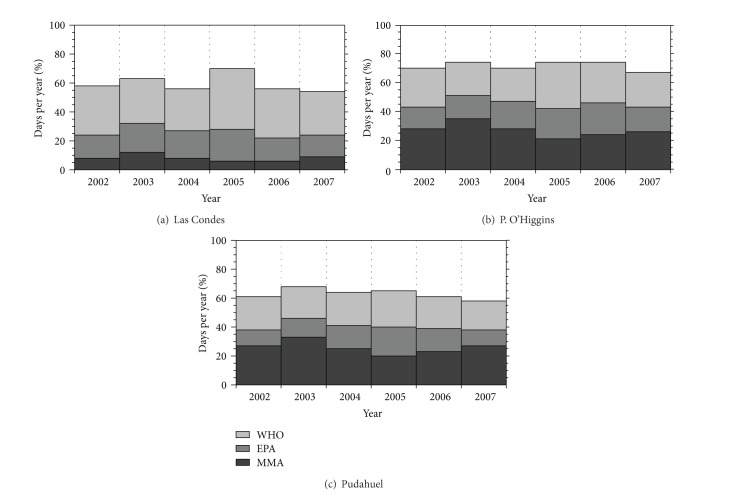
Percentage of days per year from 2002 to 2007 in which the concentration was greater than the threshold of the World Health Organization (WHO), the United States Environmental Protection Agency (EPA), and the Chilean Ministry of the Environment (MMA) standards. Label of the stations in Figure 1. Las Condes: C Label, P. O'Higgins: O Label, and Pudahuel: P Label.

Figure 6 shows the percentage of the days of the years during the study period (2002–2007) in which the PM_2.5_ concentration was greater than the 24-hour WHO guideline (25 *μ*g/m^3^) [36] and EPA (35 *μ*g/m^3^) [35] and MMA (50 *μ*g/m^3^) [34] standards. For 236 ± 24 days (65 ± 6.5%), the concentration was greater than the WHO guideline. For the EPA standard, the number of days for which the concentration was greater than the limit was 136 ± 33 (37 ± 9.1%). For the Chilean standard, the number of days for which the concentration was greater than the limit was 74 ± 36 days or 20 ± 10%. Moreover, the total carbonaceous fraction recorded in Pudahuel and Parque O'Higgins station exceeds by itself the daily standard of WHO in 74 ± 12 and 68 ± 23 days per year between 2002 and 2007 which show the significance of this fraction in the air pollution of SMA.

From these results, we can conclude that the residents of SMA are exposed to high levels of PM_2.5_ and that these particles can affect health over the long and short term.

## 4. Conclusions and Summary

The seasonal trends of PM_2.5_, EC, and OC concentrations in SMA and an analysis of the variance indicated that there were significant differences between cold seasons (March to August) and warm seasons (September to February). This difference is primarily due to the occurrence of adverse weather conditions for ventilation of the basin and to increased fuel consumption for heating. The same analysis of variance demonstrated that there was no statistically significant variability between years for any parameter studied, thereby indicating that the concentrations of these pollutants were maintained throughout 2002–2007 despite the exposure of inhabitants of SMA to critical levels of air pollution. In turn, diurnal variation during cold periods yielded two concentration peaks of PM_2.5_, OC, and TC that extended overnight and in the early hours of the morning, with the exception of the Las Condes station, at which there was an accumulation throughout the day and no peak in the morning. During warm periods, only a morning peak, which is the product of rush-hour traffic, was observed.

Bivariate polar plots showed peaks concentrations of PM_2.5_, OC, and EC related to a low wind speed and/or calm winds during cool periods. In general the air pollution events occur mainly due to local contributions and for transport phenomena associated with valley-mountain breeze observed in the city.

On average, in the city of Santiago, SOC represents 29% of total OC. In cold periods, this proportion may reach an average of 38% (for Pudahuel station), thereby suggesting that the formation processes of secondary organic particles must be incorporated into strategies for improving air quality in the forecasting model of air pollution in SMA.

Finally, a comparison of the results with the air quality standards for PM_2.5_ indicated that the total carbonaceous fraction alone exceeded the WHO (10 *μ*g/m^3^) and US-EPA (15 *μ*g/m^3^) standards during the cold seasons each year, thereby exposing the population of the city of Santiago to increased risk of acute respiratory and cardiovascular diseases.

## Figures and Tables

**Table 1:** Locations of the stations in the MACAM-2 network in the SMA used in this study.

Label^‡^	Station	Latitude (S)			Longitude (W)			Altitude (m)*
C	Las Condes	33°	22′	26′′	70°	31′	21′′	811
O	Parque O'Higgins	33°	27′	40′′	70°	39′	29′′	562
P	Pudahuel	33°	26′	06′′	70°	44′	52′′	553

^‡^Labels are according to Figure 1. *Above sea level.

**Table 2:** Statistical hourly mass concentrations of PM_2.5_, OC, EC, and TCA in *μ*g m^−3^ at the three sampling sites.

Period	PM_2.5_	OC	EC	OC/EC	TCA	TCA/PM_2.5_
	Las Condes Station^c^					
All	27 ± 17	8.6 ± 5.3	2.0 ± 1.8	6.1 ± 5.0	12 ± 6.8	0.48 ± 0.19
Cool^a^	32 ± 21	11 ± 6.1	2.7 ± 2.1	5.9 ± 5.8	15 ± 7.9	0.49 ± 0.19
Warm^b^	22 ± 11	6.3 ± 2.6	1.3 ± 1.0	6.3 ± 3.9	9.2 ± 3.4	0.47 ± 0.19

	P. O'Higgins Station^c^					
All	35 ± 27	11 ± 9.3	4.2 ± 5.3	4.8 ± 4.3	17 ± 14	0.50 ± 0.63
Cool^a^	47 ± 33	16 ± 11	6.6 ± 6.3	3.4 ± 2.5	24 ± 16	0.57 ± 0.85
Warm^b^	23 ± 12	5.5 ± 2.8	1.5 ± 1.5	6.2 ± 5.2	8.6 ± 3.9	0.41 ± 0.18

	Pudahuel Station^c^					
All	34 ± 31	11 ± 13	3.6 ± 5.8	6.0 ± 6.3	17 ± 18	0.47 ± 0.19
Cool^a^	47 ± 39	18 ± 16	6.1 ± 7.5	4.9 ± 5.2	26 ± 23	0.52 ± 0.19
Warm^b^	21 ± 12	5.4 ± 3.7	1.3 ± 1.4	7.1 ± 7.0	8.3 ± 4.8	0.42 ± 0.18

	Average all stations					
All	32 ± 26	11 ± 10	3.4 ± 5.0	5.6 ± 5.3	16 ± 15	0.48 ± 0.41
Cool^a^	42 ± 31	15 ± 11	5.1 ± 5.3	4.7 ± 4.5	22 ± 16	0.52 ± 0.41
Warm^b^	22 ± 12	5.7 ± 3.0	1.4 ± 1.3	6.6 ± 5.4	8.7 ± 4.1	0.43 ± 0.18

^a^Cool seasons are winter and autumn (March to August).

^b^Warm seasons are spring and summer (September to February).

^c^Label of the stations in Figure 1. Las Condes: C Label, P. O'Higgins: O Label, and Pudahuel: P Label.

**Table 3:** One-way ANOVA of seasonal and annual variability in PM_2.5_, OC, and EC concentrations at the sites studied.

Station	Comparison criteria	PM_2.5_		OC		EC	
		*F*-test	*P* value	*F*-test	*P* value	*F*-test	*P* value
Las Condes^a^	Between years^b^	0.92	0.54	2.85	0.17	1.95	0.27
	Between seasons^c^	91.5	2 × 10^−4^	43.3	0.003	13.0	0.02
P. O'Higgins^a^	Between years	0.73	0.63	0.91	0.54	0.99	0.51
	Between seasons	74.4	3 × 10^−4^	137	8 × 10^−5^	50.9	8 × 10^−4^
Pudahuel^a^	Between years	0.69	0.65	1.28	0.40	1.24	0.41
	Between seasons	253	2 × 10^−5^	79.1	2 × 10^−4^	25.7	3 × 10^−3^
Average all stations	Between sites	9.56	0.001	4.18	0.029	6.10	0.008

^a^Label of the stations in Figure 1. Las Condes: C Label, P. O'Higgins: O Label, and Pudahuel: P Label.

^b^From 2002 to 2007.

^c^Between cool and warm seasons.

**Table 4:** OC versus EC linear correlation in PM_2.5_ for the stations studied in the period from 2002 to 2007. *a* is the slope, *b* (adim) is the intercept (*μ*g/m^3^), *R* ^2^ is the square of the correlation coefficient, and *n* is the number of data pairs.

Site	Period	OC(*y*) = *a* + *b*EC(*x*)			
		Intercept, *a* (*μ*g m^−3^)	Slope, *b* (adim)	*R* ^2^	*n*
Las Condes^a^	Cool	4.19 ± 0.05	2.48 ± 0.01	0.68	12907
	Warm	3.74 ± 0.03	1.93 ± 0.02	0.48	11853
P. O'Higgins^a^	Cool	6.60 ± 0.07	1.35 ± 0.01	0.64	18745
	Warm	3.63 ± 0.02	1.30 ± 0.01	0.47	17165
Pudahuel^a^	Cool	7.04 ± 0.09	1.80 ± 0.01	0.62	17882
	Warm	2.98 ± 0.06	1.90 ± 0.01	0.49	19603

^a^Label of the stations in Figure 1. Las Condes: C Label, P. O'Higgins: O Label, and Pudahuel: P Label.

**Table 5:** OC versus EC minimum linear correlation in PM_2.5_ for the stations studied in the period from 2002 to 2007. *a* is the slope, *b* (adim) is the intercept (µg/m^3^), *R* ^2^ is the square of the correlation coefficient, and *n* is the number of data pairs.

Site	Period	OC(*y*) = *a* + *b*EC(*x*)			
		Intercept, *a* (*μ*g m^−3^)	Slope, *b* (adim)	*R*_min⁡_^2^	*n*_min⁡_
Las Condes^a^	Cool	3.5 ± 0.2	1.92 ± 0.05	0.72	627
	Warm	2.7 ± 0.2	1.31 ± 0.06	0.51	538
P. O'Higgins^a^	Cool	5.8 ± 0.3	1.20 ± 0.02	0.74	879
	Warm	2.8 ± 0.2	1.15 ± 0.03	0.54	805
Pudahuel^a^	Cool	5.0 ± 0.4	1.48 ± 0.01	0.75	877
	Warm	1.7 ± 0.1	1.43 ± 0.04	0.62	801

^a^Label of the stations in Figure 1. Las Condes: C Label, P. O'Higgins: O Label, and Pudahuel: P Label.

**Table 6:** Average inorganic and organic fractions (as percentages), total carbon (TC) incorporated into the PM_2.5_, and organic (OC) and elemental carbon (EC) fractions as percentages of the total carbon (TC) in the period from 2002 to 2007 at the three stations studied in the cool and warm seasons.

Site	Period	PM_2.5_ (*μ*g m^−3^)	Inorganic fraction (IF%)	Carbon fraction (CF%)	TOC (*μ*g m^−3^)	POC (%)	SOC (%)
Las Condes^a^	Cool	32.0 ± 20.6	57.9	42.1	10.8 ± 6.1	70	30
	Warm	21.8 ± 11.3	65.0	35.0	6.3 ± 2.6	75	25
P. O'Higgins^a^	Cool	47.5 ± 32.9	53.3	46.7	15.6 ± 10.5	73	27
	Warm	23.2 ± 11.6	69.9	30.1	5.5 ± 2.8	76	24
Pudahuel^a^	Cool	46.6 ± 39.0	48.1	51.9	18.1 ± 16.4	62	38
	Warm	21.0 ± 11.6	68.1	31.9	5.4 ± 3.7	71	29

^a^Label of the stations in Figure 1. Las Condes: C Label, P. O'Higgins: O Label, and Pudahuel: P Label.
